# Elite Rugby Players have Unique Morphological Characteristics of the Hamstrings and Quadriceps Femoris Muscles According to their Playing Positions

**DOI:** 10.2478/hukin-2022-0039

**Published:** 2022-09-08

**Authors:** Raki Kawama, Masamichi Okudaira, Seigo Shibata, Tatsuya Shimasaki, Hirohiko Maemura, Satoru Tanigawa

**Affiliations:** 1 Graduate School of Health and Sports Science, Doshisha University, Kyoto, Japan; 2 Research Fellow of Japan Society for the Promotion of Science, Tokyo, Japan; 3 Faculty of Health and Sports Sciences, University of Tsukuba, Ibaraki, Japan; 4 Insutitute of Health and Sports Sciences, University of Tsukuba, Ibaraki, Japan

**Keywords:** muscle size, forward, backs, ultrasonography

## Abstract

Rugby is a popular sport requiring high-intensity and maximal speed actions. Numerous studies have demonstrated that physical performance variables, such as strength, sprinting, and jumping, are different between the forwards and backs. However, there is little information about muscle morphological characteristics specific for each rugby playing position. This study aimed to clarify the morphological characteristics of the thigh muscles in forwards and backs. Ultrasound images were obtained from the proximal, middle, and distal regions of the thigh. Then, the anatomical cross-sectional areas of particular muscles in the hamstrings and quadriceps femoris were calculated for seven forwards, seven backs, and ten non-athletes. The anatomical cross-sectional areas were normalised by the two-third power of lean body mass, and the normalised values of the three regions were averaged as that of the individual muscle. In the hamstrings, the normalised anatomical cross-sectional areas of the biceps femoris long head were significantly greater in forwards than in non-athletes, whereas those of the semitendinosus were significantly greater in backs than in non-athletes. Furthermore, in the quadriceps femoris, the normalised anatomical cross-sectional areas of the rectus femoris and vastus intermedius were significantly greater in forwards than in backs and non-athletes. These results suggest that forwards have great muscularity of the biceps femoris long head and vastus intermedius which can generate large force, whereas backs possess great muscularity of the semitendinosus which can generate high contraction velocity. These findings allow coaches to design more effective training programs according to particular rugby playing positions.

## Introduction

Rugby, one of the world’s most popular sports, requires various high-intensity activities such as sprinting, jumping, and contact actions. The playing positions in rugby are mainly divided into two groups, i.e., “forwards” and “backs”, based on their physical demands in competitive activities. Forwards frequently participate in scrums, rucks, and mauls with great contact (+60% than backs) (Cunniffe et al., 2009) to maintain the possession of the ball during a game. Conversely, backs usually cover a greater distance (+35.4% than forwards) (Cahillet al., 2013) and enter faster speed running at 20 km/h (+50% than forwards) (Cunniffe et al., 2009) to carry the ball to the goal line during a game. Because of these positional differences in physical demands, several studies have reported significant differences in maximal muscular strength, 30-m sprint time, and agility between forwards and backs (Brown et al., 2014; La Monica et al., 2016; Zabaloy et al., 2020). Meanwhile, physical performance variables are affected by physiological factors, such as neural activation, histochemical, and morphological characteristics of the muscle. Among them, the morphological characteristics, especially muscle size, have been demonstrated to be closely associated with muscular strength and sprint ability (Abdi et al., 2019; Blazevichet al., 2009; Ema, et al., 2018; Fukunaga et al., 2001). Hence, clarifying rugby players’ morphological characteristics could be a beneficial approach to identify which muscle is vital for achieving the required movements in forwards and backs. Nevertheless, there is little research investigating muscle morphological characteristics of rugby players at each position.

In rugby, thigh muscles, including the hamstrings and the quadriceps femoris, play an important role in sprinting, jumping, and contact actions. Specifically, each of the muscle groups mainly works as hip and knee extensors to powerfully push the ground during the rugby’s competitive activities. In general, the constituent muscles of a muscle group are considered to work together; however, it should be emphasized that particular muscles of the hamstrings and quadriceps femoris have different morphological and functional features. For example, in the hamstrings, the biceps femoris long head (BFlh) and the semimembranosus (SM) possess large physiological cross-sectional area, whereas the semitendinosus (ST) and the biceps femoris short head (BFsh) have long muscle fibres (Woodley and Mercer, 2005). Furthermore, in the quadriceps femoris, the vastus lateralis (VL) and the vastus intermedius (VI) possess a larger physiological cross-sectional area compared to that of the rectus femoris (RF) and the vastus medialis (VM) (Massey et al., 2015). These morphological features suggest that BFlh, SM, VL, and VI contribute to the generation of large forces, while ST and BFsh contribute to the generation of high contraction velocities in competitive movements (Lieber and Friden, 2000). It has been shown that daily competitive activities strongly affect muscularity in athletes (Ema et al., 2018; Hoshikawa et al., 2010). Since forwards frequently take part in contact actions which require lower joints to produce great torque, while backs usually participate in sprinting and jumping which require high contraction velocities of lower-limb muscles, the morphological characteristics of thigh muscles may differ between forwards and backs. A previous study reported that forwards had a larger muscle size of VL compared to that of backs (La Monica et al., 2016). However, the previous study did not examine the morphological characteristics of the other thigh muscles. Hence, it remains unknown whether the morphological characteristics of the thigh muscles are specific to playing positions.

Therefore, this study aimed to clarify the morphological characteristics of the thigh muscles (i.e., particular muscles of the hamstrings and quadriceps femoris) in forwards and backs in male collegiate rugby players. We hypothesised that forwards would show greater muscularity of BFlh, SM, VL, and VI which are designed to generate large force, whereas backs would exhibit greater muscularity of ST and BFsh which are suitable to generate high contraction velocity. This investigation attempted to provide a deeper understanding of morphological differences between forwards and backs, what would allow coaches to design more effective training programs according to specific rugby playing positions.

## Methods

### Participants

Fourteen male collegiate rugby athletes (age: 20.6 ± 1.4 years, body height: 176.7 ± 6.2 cm, body mass: 89.8 ± 12.5 kg) and 10 non-athletes (age: 23.3 ± 0.5 years, body height: 172.4 ± 7.1 cm, body mass: 65.8 ± 8.0 kg) participated in this study. The rugby athletes’ team stood third in the All-Japan University Rugby Football Championship two years ago. Overall, they had an average of 11.9 ± 3.7 years of competitive rugby experience. Additionally, nine of them belonged to the Japanese national rugby team which includes players under 18 and/or 20 years. They were grouped in this study as either forwards (n = 7) or backs (n = 7) based on their competitive experience. Both groups normally participated in seven weekly training sessions (five rugby sessions and two sessions of resistance training, and sprint running) and underwent identical resistance training programs. There were no significant positional differences in age (forwards: 20.6 ± 1.1 years, backs: 20.6 ± 1.7) or rugby experience (forwards: 11.4 ± 4.0 years, backs: 12.3 ± 3.6). Meanwhile, non-athletes did not engage in conventional sports activities for at least two years. None of the participants had musculoskeletal injuries over the past two years. All participants were carefully informed of the purpose and procedures and gave written informed consent prior to participation in the study. All experimental protocols were approved by the research ethics committee of the authors’ institution and based on the Declaration of Helsinki.

### Experimental design

To achieve the goal of this study, anatomical cross-sectional areas (ACSAs) of particular hamstring (BFlh, BFsh, ST, and SM) and quadriceps muscles (RF, VL, VM, and VI) were determined from the proximal, middle, and distal regions using an extended field of view ultrasonography. The ACSAs of the individual muscles were then compared among forwards, backs, and non-athletes.

### Procedures

**Figure 1:**
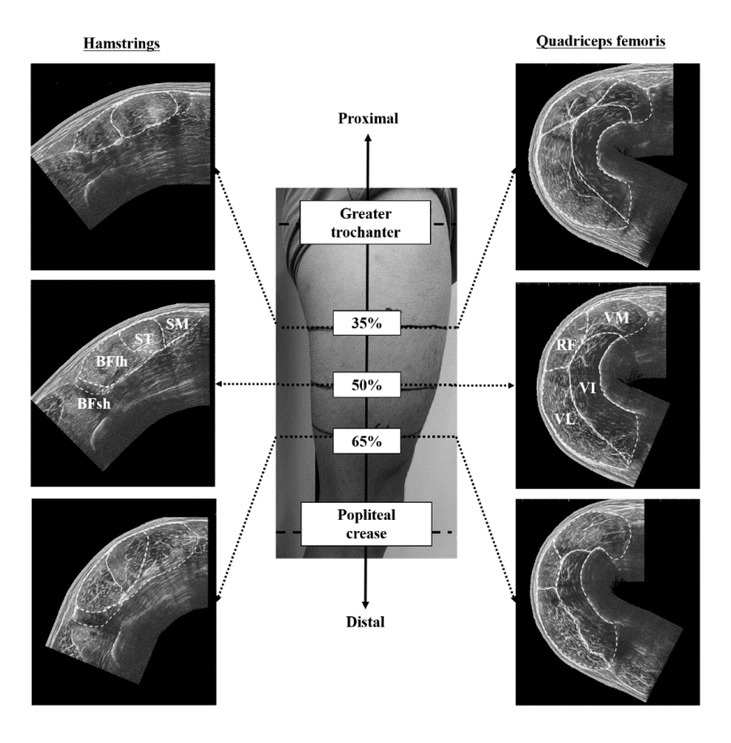
Measurement locations of the anatomical cross-sectional area and example of the ultrasound images in the thigh muscles. The white broken line indicates the outline of individual muscles of the hamstring and quadriceps femoris. BFlh, biceps femoris long head; BFsh, biceps femoris short head; ST, semitendinosus; SM, semimembranosus; RF, rectus femoris; VL, vastus lateralis; VM, vastus medialis; VI, vastus intermedius.

The thigh length (i.e., the distance between the greater trochanter and popliteal crease) was measured on the dominant leg. If the participant had a history of severe injuries (e.g., hamstring strain injury of grade 3 and/or anterior cruciate ligament injury), the non-injured leg was used for the measurements. The locations at 35%, 50%, and 65% of the thigh length were marked as an axial perpendicular line and defined as the proximal, middle, and distal regions, respectively. For ACSA measurements of the hamstrings, participants were required to lie prone on a massage bed at 0° of knee flexion. For ACSA measurements of the quadriceps femoris, they were asked to lay supine on the bed with 30° of knee flexion because the edges of VL and VM were in contact with the bed at 0° of knee flexion. Ultrasound images were acquired from the proximal, middle, and distal regions of the thigh-length using an extended field of view ultrasonography (EUP-L53, 6–10 MHz, 64 mm field of view, Noblus, Hitachi, Tokyo, Japan) (Figure 1). After applying transmission gel between the skin and the scanner, the probe was moved slowly and carefully on the transverse plane perpendicular to the femur, from the medial to the lateral side of the thigh in each region. A leather belt was attached to the participant’s thigh with minimal pressure to keep the probe perpendicular to the femur. All ultrasound measurements were performed by the same investigator with more than three year experience in musculoskeletal ultrasonography.

From each ultrasound image, the ACSA of particular hamstring (BFlh, ST, and SM) and quadriceps femoris muscles (RF, VL, VM, and VI) were analysed twice at the proximal, middle, and distal regions of the thigh using Image J software (version 1.52, National Institutes of Health, Bethesda, MD, USA). The ACSAs of BFsh were calculated twice in only the middle and distal regions, as the muscle is a monoarticular muscle which does not cross the hip joint. The average of the two values was used as the ACSA. For normalization of ACSAs, lean body mass was measured using the InBody 720 Body Composition Analyzer (Biospace Corporation, Seoul, Korea). The ACSAs were normalised by the two-third power of lean body mass (nACSA) (Hoshikawa et al., 2010). In particular muscles, nACSAs of two or three regions were averaged as the nACSA of each muscle. Although the measurement validity of this ultrasound technique has been already verified in some studies (Kositsky et al., 2020; Noorkoiv et al., 2010), we ensured test-retest reliability by acquiring two ultrasound images from each region of the thigh. The intraclass correlation coefficients of ACSAs of the two ultrasound images were 0.815–0.999. These values could be interpreted as “almost perfect” (Landis and Koch, 1997).

### Statistical Analyses

All data are presented as means and standard deviation. In this study, each group had a small sample size, and the distribution of the data was partly non-Gaussian. Thus, the Kruskal-Wallis test was used to determine significant differences in the ACSA among the groups regarding particular muscle groups and muscles. If necessary, the Mann-Whitney *U* test was performed to identify which pairs indicated significant differences in the ACSA. The level of significance was set at 0.05. This was modified using the Benjamini and Hochberg method for multiple tests (Benjamini and Hochberg, 1995). Cohen’s *d* was used to calculate the effect sizes. The values of 0.2, 0.5, and 0.8 were interpreted as small, medium, and large effects, respectively (Cohen, 1988). All statistical analyses were performed using SPSS software (version 25.0, IBM Corporation, Armonk, USA).

## Results

**Figure 2:**
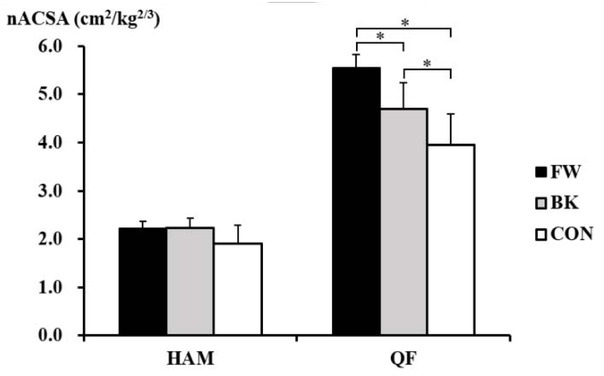
The normalised anatomical cross-sectional area of the hamstrings and quadriceps femoris in forwards (FW), backs (BK), and non-athletes (CON). *Significant difference between the groups. HAM, hamstrings; QF, quadriceps femoris.

The Kruskal-Wallis test revealed significant inter-group differences in the nACSA in the quadriceps femoris (*p* = 0.001), but not in the hamstrings (*p* = 0.069) (Figure 2). The post hoc test showed that the nACSA of the quadriceps femoris was significantly greater in forwards than in backs (*p* = 0.003, *d* = 1.95) and non-athletes (*p* = 0.020, *d* = 3.23). This was also larger in backs than in non-athletes (*p* = 0.019, *d* = 1.26).

**Figure 3:**
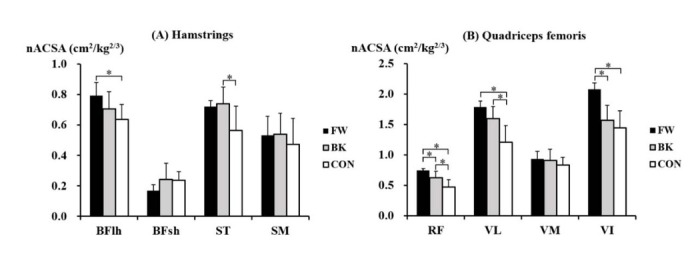
The normalised anatomical cross-sectional area of particular muscles of the (A) hamstrings, and (B) quadriceps femoris in forwards (FW), backs (BK), and non-athletes (CON). *Significant difference between the groups. BFlh, biceps femoris long head; BFsh, biceps femoris short head; ST, semitendinosus; SM, semimembranosus; RF, rectus femoris; VL, vastus lateralis; VM, vastus medialis; VI, vastus intermedius.

Within the hamstrings, there were significant inter-group differences in the nACSA in BFlh (*p* = 0.029) and ST (*p* = 0.026) (Figure 3A). The post-hoc test showed that the nACSA of BFlh was significantly greater in forwards than in non-athletes (*p* = 0.001, *d* = 0.91), while the nACSA of ST was significantly greater in backs than in non-athletes (*p* = 0.015, *d* = 1.27). Within the quadriceps femoris, significant inter-group differences in the nACSA were found in RF (*p* = 0.001), VL (*p* = 0.002), and VI (*p* = 0.002) (Figure 3B). The nACSA of RF was also significantly greater in forwards than in backs (*p* = 0.026, *d* = 1.54) and non-athletes (*p* < 0.001, *d* = 3.16) and was also larger in backs than in non-athletes (*p* = 0.019, *d* = 1.33). In VL, both forwards (*p* = 0.002, *d* = 2.82) and backs (*p* = 0.010, *d* = 1.61) had a significantly greater nACSA than that of non-athletes, whereas forwards had a significantly larger nACSA of VI compared to that of backs (*p* = 0.002, *d* = 2.67) and non-athletes (*p* = 0.003, *d* = 2.99).

## Discussion

The present results indicate that the nACSA of BFlh is greater in forwards than in non-athletes, whereas the nACSA of ST is greater in backs than in non-athletes. Additionally, nACSAs of RF and VI are larger in forwards than in backs and non-athletes. These results partly support our hypothesis and suggest that forwards have great muscularity of BFlh and VI which can generate large force, whereas backs possess great muscularity of ST which can generate high contraction velocity. To date, positional differences in muscle size have been examined only in VL; however, no information on the morphological characteristics of the other thigh muscles have been provided (La Monica et al., 2016). Thus, to the best of our knowledge, this is the first study to demonstrate that morphological characteristics of thigh muscles are specific to rugby playing positions.

Compared to backs and non-athletes, forwards had greater muscularity of BFlh, VI, and RF. During games and daily competitive training, forwards frequently participate in contact actions such as scrums, rucks, and mauls. During these movements, each of the muscles in the hamstrings and quadriceps femoris would need to produce great extension torques of the hip and knee joints to push the ground powerfully. Among the hamstring muscles, physiological cross-sectional areas are larger in BFlh and SM than in BFsh and ST, suggesting that the former muscles contribute more to generation of large forces (Woodley and Mercer, 2005). Moreover, BFlh has the longest moment arm for hip extension across all hip joint angles (Arnold et al., 2000; Visseret al., 1990). Since the magnitude of muscle torque is the product of muscle force and its moment arm, BFlh is likely to have a great potential for generating hip extension torque in the hamstrings. In fact, the normalised electromyographic activity was reported to be higher in BFlh than in ST and SM during hip extension exercise (Bourne et al., 2017). The magnitude of muscle activation during an exercise is associated with the muscle hypertrophy pattern following a long-term training intervention (Wakahara et al., 2013); therefore, the competitive movements which require large hip extension torque may lead to greater BFlh muscularity in forwards.

Regarding greater muscularity of VI in forwards, its moment arm for knee extension is similar to that of the other quadriceps femoris muscles across all knee joint angles (Visser et al., 1990). However, VI possesses the largest physiological cross-sectional area, which could provide approximately one-third of the force-generating capacity of the quadriceps femoris (Akima et al., 2007; Massey et al., 2015). Moreover, an electromyographic study suggested that VI contributed up to 50% of the knee extension torque during submaximal isometric contraction (Zhang et al., 2003). Thus, VI primarily contributes to the generation of knee extension torque in competitive activities with great contacts. In addition, forwards in this study also showed greater muscularity of RF, despite having the least physiological cross-sectional area among the quadriceps femoris muscles (Massey et al., 2015). Its underlying mechanism is unclear; however, the biarticular nature of RF may be implicated. In the quadriceps femoris, only RF is a biarticular muscle crossing knee and hip joints. It has been shown that the fascicle shortening velocity of the biarticular muscle is affected by both the proximal and distal joint angles. Specifically, the estimated muscle contraction velocity of RF was lower than that of the monoarticular vastus muscles during leg extension (Gregoire et al., 1984). In general, force-generating capacity depends on muscle contraction velocity; thus, RF could also greatly contribute to the torque production during knee extension, especially in low angular velocity movements such as scrums, rucks, and mauls.

The present results indicate greater muscularity of ST in backs, who usually perform high-speed running in games and daily competitive training. It is known that ST has the longest fascicle length among the hamstring muscles (Woodley and Mercer, 2005). Since fibre length is a determinant of the maximal shortening velocity of the muscle (Bodine et al., 1982), ST could generate a large torque in high-velocity movements, such as sprinting and jumping (Lieber and Friden, 2000). During maximum speed sprinting, normalised electromyographic activation was greater in ST than in BFlh (Higashihara et al., 2018). In addition, the muscle volume of ST in sprinters was 54% larger than that in non-athletes, whereas these differences were only 20–26% for the other hamstring muscles (Handsfield et al., 2017). Based on these findings, competitive activities which require high-speed running result in greater muscularity of ST in backs.

Contrary to a previous study which showed greater muscularity of VL in forwards (La Monica et al., 2015), no positional differences in the nACSA of VL were demonstrated in the present study. This discrepancy in results can be explained by the fact that ACSAs were represented as absolute values in the previous study (La Monica et al., 2015), while these were expressed as normalised values in our study. Our results add new information that relative muscle size of VL to lean body mass is not position-specific in rugby players. Furthermore, the mean rugby experience was substantially longer in athletes included in this study (mean of 11.9 ± 3.7 years) than that in the previous research (mean of 2.2±2.2 years; La Monica et al., 2015). These facts provide a possibility that long-term competitive training of rugby may not necessarily induce specific morphological adaptation of VL according to the playing position.

A deep understanding of athletes’ morphological characteristics is essential to design an effective training program which enhances sports performance. However, in both training and research fields, the morphologies of rugby players have only been understood roughly by thigh length, thigh circumference, and body mass (La Monica et al., 2016; Zabaloy et al., 2020). Meanwhile, we provided possible evidence on which muscle is important for achieving the required movements in forwards and backs. In the present study, forwards had great muscularity of BFlh, whereas backs had great ST muscularity. A recent study showed that the transverse relaxation time of BFlh greatly changed after hip extension exercise (e.g., stiff-leg deadlift), while that of ST significantly changed after knee flexion exercise (e.g., Nordic hamstrings) ([Bibr j_hukin-2022-0039_ref_025][Bibr j_hukin-2022-0039_ref_026]). Thus, each of the hip extension and knee flexion exercises would be recommended for forwards and backs to efficiently improve their physical performances by developing a specific muscle within the hamstrings and quadriceps femoris. The present study also showed that forwards had greater muscularity of RF and VI than backs. It was reported that electromyographic activation in all regions of RF was greater during knee extension than during hip flexion (Miyamoto et al., 2012). Hence, knee extension exercises are more suitable for forwards to strengthen both RF and VI. Taken together, coaches need to carefully design training programs considering the different morphological characteristics between forwards and backs.

This study has limitations which should be addressed. Muscle morphological differences between forwards and backs may also be influenced by genetic predispositions and resistance training exercise routines. However, rugby players in our study had an average of 11.9 ± 3.7 years of competitive rugby experience. Moreover, they routinely performed identical resistance training programs at a similar frequency. Thus, the unique morphological characteristics of each rugby position could be mainly attributed to their daily competitive activities.
